# A Rare Case of Benzocaine-Induced Methemoglobinemia

**DOI:** 10.7759/cureus.19259

**Published:** 2021-11-04

**Authors:** Diva C Maraj, Ronda Barak-Norris, Melody Dankha

**Affiliations:** 1 Allergy and Immunology, St. Joseph Mercy Oakland Hospital, Pontiac, USA; 2 Allergy and Immunology, Beaumont Hospital, Royal Oak, USA; 3 Allergy and Immunology, Ascension Providence Hospital, Southfield, USA; 4 Research, St. Joseph Mercy Oakland Hospital, Pontiac, USA

**Keywords:** chocolate-colored blood, methemoglobinemia, methylene blue, hypoxia, benzocaine

## Abstract

Methemoglobinemia is a severely dangerous condition that can be induced by congenital mutations or can be acquired. One of the ways to acquire methemoglobinemia is by using topical anesthetics during procedures, such as nasogastric (NG) tube placement, transesophageal echocardiogram (TEE), esophagogastroduodenoscopies (EGD), and during endoscopic retrograde cholangiopancreatography (ERCP).

Herein, we present the case of a 35-year-old lady who presented to the hospital for an initial hysterectomy. However, due to topical anesthetic use during an NG tube placement, she developed methemoglobinemia. She then developed hypoxia, an altered mental status, and had elevated methemoglobinemia levels. She denied any previous episode of methemoglobinemia and had no family history of the condition as well. She was rapidly given methylene blue, which resolved her symptoms and induced normal methemoglobin levels on subsequent arterial blood gas analysis. Those who are unknowingly susceptible to developing methemoglobinemia and receive anesthetics during hospital procedures are at risk for serious adverse effects and clinical deterioration if not treated correctly. Therefore, it is important to recognize the clinical signs of methemoglobinemia as soon as they appear and have the required treatment readily available, as any delay could result in dangerous consequences for the patient.

## Introduction

Methemoglobinemia is a severely dangerous condition, which results from the iron in hemoglobin converting to the ferric state [1]. This can result in a patient's death if not treated promptly. It can be induced by congenital mutations or it can be acquired. One of the ways to acquire methemoglobinemia is by using topical anesthetics, such as nitrates, benzocaine, and lidocaine [1]. At first, methemoglobinemia can appear asymptomatic. However, symptoms will quickly develop, such as unresponsiveness, hypoxia, and trouble breathing [1]. The first case of benzocaine-induced methemoglobinemia was reported in 1977 [2]. Topical anesthetics used during procedures, such as nasogastric (NG) tube placement, transesophageal echocardiogram (TEE), esophagogastroduodenoscopy (EGD), and during endoscopic retrograde cholangiopancreatography (ERCP), can induce methemoglobinemia [3]. It occurs in approximately 3.5 of 10,000 cases of reported methemoglobinemia [4-5]. Those who are unknowingly susceptible to developing methemoglobinemia and receive anesthetics during hospital procedures are at risk for serious adverse effects and clinical deterioration if not treated correctly.

## Case presentation

**Figure 1:**
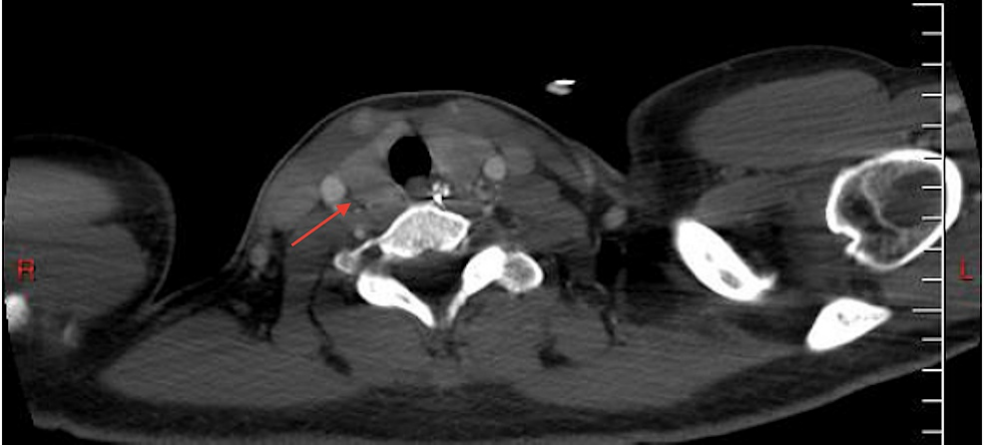
Computed tomography of the abdomen and pelvis, indicating fibroids (red arrow)

**Figure 2:**
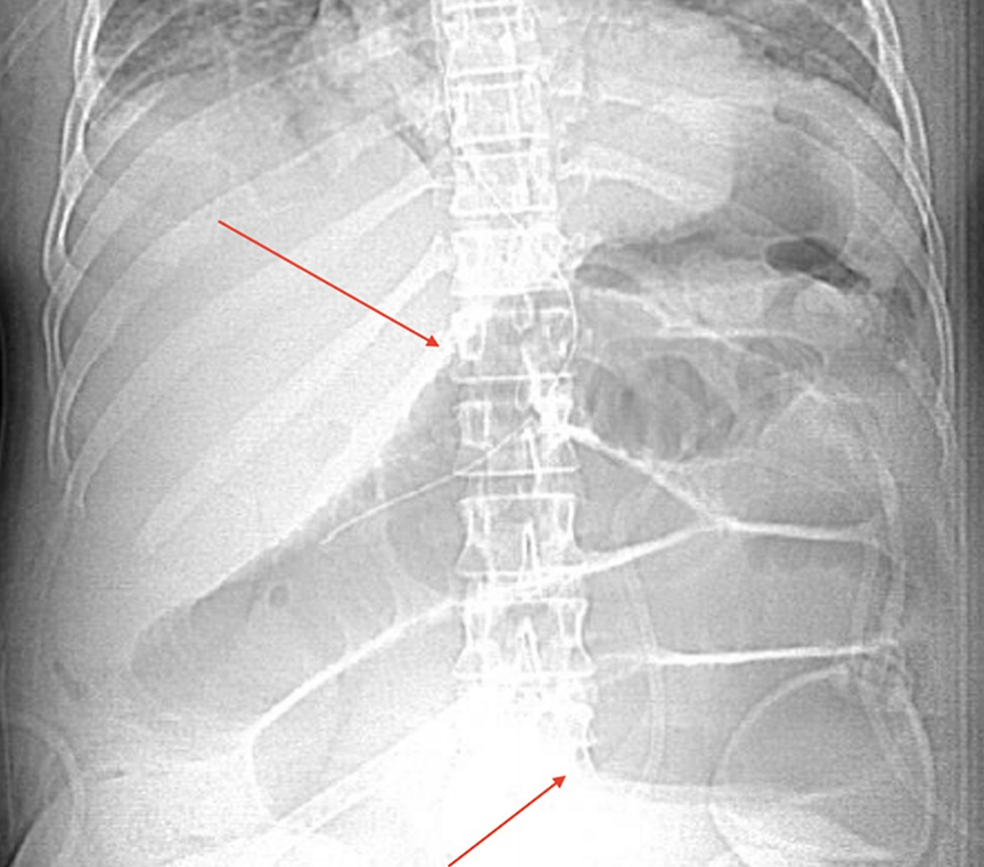
Abdominal x-ray indicating a postoperative ileus (red arrows)

**Figure 3:**
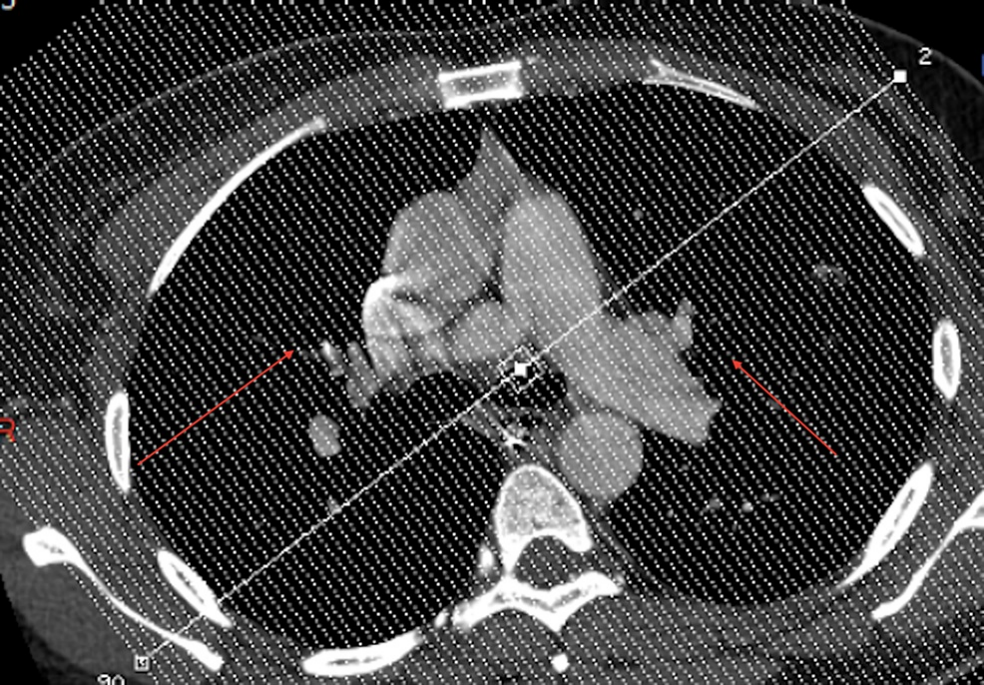
Computed tomography of the patient's chest indicating bilateral pulmonary infiltrates (red arrows)

**Figure 4:**
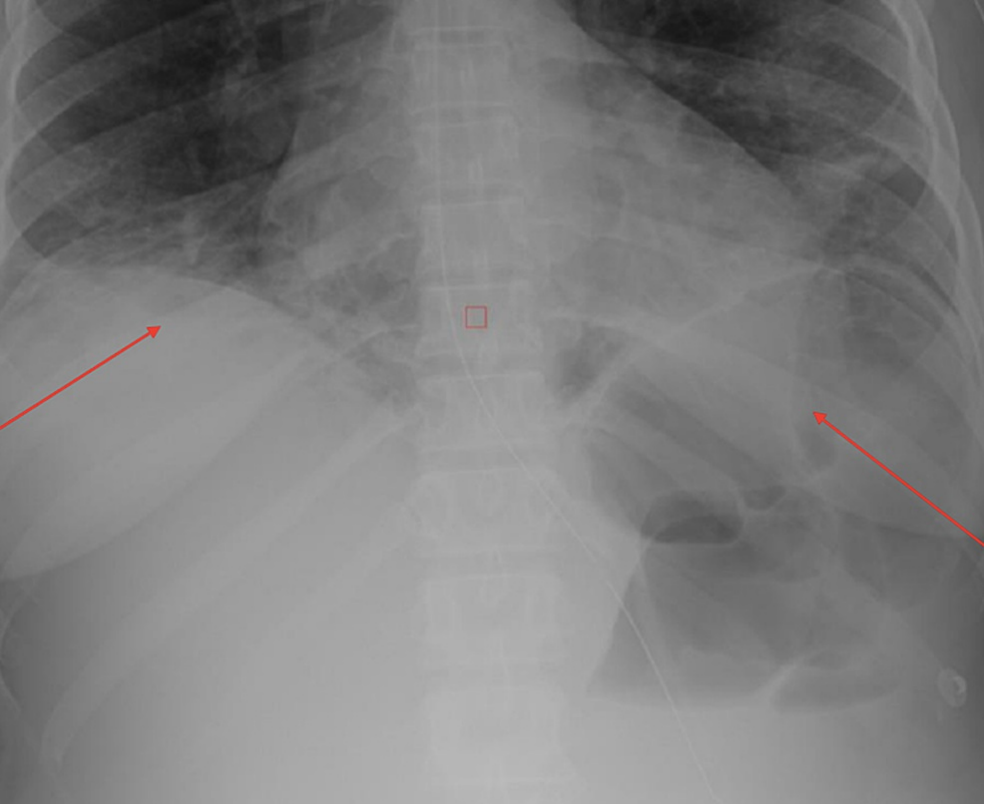
Chest x-ray of the patient indicating pneumonia or aspiration (red arrows)

A 35-year-old African American woman with a past medical history of enlarging fibroids, primary arterial hypertension, essential thrombocythemia (JAK2^V617F^-positive), Raynaud’s phenomenon, and alopecia was admitted to the hospital for a hysterectomy due to the patient’s menorrhagia. The patient was a former smoker who quit in late 2014, drank alcohol occasionally, and denied any illicit drug use. She was 5 feet 4 inches tall, with a body mass index (BMI) of 26.78 kg/m^2^. Her physical examination was unremarkable, except for abdominal distension secondary to uterine fibroids which extended 3 cm above the umbilicus (Figure 1). There was also tenderness of the uterus upon palpation. On admission to the hospital, she underwent an abdominal hysterectomy with bilateral salpingectomy, as well as an abdominoplasty. The patient was noted to have a low hemoglobin level of 6.8 g/dL after the procedure. A postoperative computed tomography (CT) scan revealed a large intra-abdominal hematoma. The patient underwent a diagnostic laparotomy with conversion to an exploratory laparotomy. She developed postoperative ileus after the exploratory laparotomy (Figure 2). A nasogastric (NG) tube was placed after benzocaine was sprayed into the oropharynx to prevent pharyngeal discomfort. The following night, the patient developed tachycardia, hypoxia (88% on room air), and a fever. She was subsequently transferred to the intensive care unit. A computed tomography angiography (CTA) of her chest showed bilateral extensive infiltrates (Figure 3). A chest x-ray also indicated bibasilar airspace opacification, due to either aspiration or pneumonia (Figure 4).

She was started on piperacillin-tazobactam and vancomycin with supplemental oxygen. Later that night, the patient was noted to have an altered mental status. Her NG tube output displayed dark chocolate-colored blood. A stat arterial blood gas analysis showed a PO_2_ level of 253 mmHg, pCO_2_ of 29.8 mmHg, and an elevated methemoglobin level of 28.9% (Table 1). The elevated methemoglobinemia was secondary to receiving benzocaine spray during her NG tube placement. The patient received one dose of methylene blue, 1 mg/kg as a 1% solution, with rapid resolution of her symptoms and normalization of methemoglobin levels to 1.1%, pCO_2_ of 32.8 mmHg, and PO_2_ of 94.2 mmHg. The patient was discharged home in stable condition after a month of observation in the hospital due to postoperative aspiration pneumonitis, sepsis, anemia, and ileus. This patient had no significant family history of methemoglobinemia, nor did she have any genetic testing looking for a congenital cause of methemoglobinemia.

**Table 1:** Patient's Methemoglobin Blood (MetHb) Levels COHb: carboxyhemoglobin; FIO_2_: fraction of inspired oxygen; HCO_3_ - bicarbonate; pCO_2_ – partial pressure of carbon dioxide; pH - potential of hydrogen; PO_2_ - partial pressure of oxygen; SO_2_ – sulfur dioxide; tHb: total hemoglobin

Date/Time	Day 1, Afternoon	Day 1, Mid-Morning	Day 1, Later That Morning	Day 1, Early Morning
Temperature (Fahrenheit)	98.6°	98.6°	98.6°	98.6°
Fi0_2_	28%	21%	50%	100%
Liter_Flow (L/min)	2	2	2	2
pH	7.38	7.42	7.45	7.46
pC0_2 _(mmHg)	37.5	32.8	30	29.8
PO_2_ (mmHg)	47.30	94.2	255	253
HC0_3_ (mEq/L)	21.7	20.7	20.5	20.8
tHb (g/dL)	7.9	8.1	8.5	8.7
S0_2_	81.7%	97%	99.8%	98.5%
COHb	1.1%	1.1%	0.1%	0
MetHb	4%	1.1%	9%	28.9%

## Discussion

Methemoglobinemia is a form of hemoglobin that has been oxidized, changing its heme iron configuration from Fe2+ to Fe3+ (ferric state) [1]. Acquired causes are from medications not limited to nitrates, dapsone, benzocaine, lidocaine and prilocaine, such as in this patient who received benzocaine during a routine NG tube insertion for a postoperative ileus. This produces a shift in the hemoglobin-oxygenation dissociation curve to the left, and further decreases oxygen delivery to tissues, resulting in functional anemia. People with acute toxic methemoglobinemia can be severely ill and can die from severe hypoxia, despite being given supplemental oxygen. After receiving benzocaine, this patient was hypoxic at 88%, wasn't responding to supplemental oxygen, and had an altered mental status. Congenital causes of methemoglobinemia include being deficient in the enzyme cytochrome b5 reductase (Cyb5R) [3]. It can also be caused by Hb M, an autosomal dominant disease, in which the reduced ferrous ion is destabilized and can be easily oxidized to the ferric state [6]. This patient denied any family history of methemoglobinemia and never had any genetic testing completed. She only reported a past medical history of essential thrombocythemia, which was stable at the time. 

Patients with methemoglobinemia can be asymptomatic [1]. Once levels exceed 20%, they can develop hypoxia, cyanosis, dyspepsia, headache, lightheadedness, fatigue, irritability, lethargy, shock, respiratory depression, coma, seizures, and altered mental status, such as in this patient [3]. Levels > 70% are typically fatal [3]. Methemoglobinemia is mainly detected through a blood gas analysis, as it has an absorbance spectrum of 631 nm [1]. Methemoglobinemia can be treated with methylene blue or ascorbic acid [1]. Methylene blue is a reducing agent through the nicotinamide adenine dinucleotide phosphate (NADPH) methemoglobin reductase pathway and in low concentrations, it is reduced to leucomethylene blue, which reacts with methemoglobin in the blood, reducing it to hemoglobin [1]. A low dose of methylene blue is given, 1 - 2 mg/kg of a 1% solution IV over five mins, and the dose can be repeated within one hour if hypoxia symptoms aren’t resolved [3]. This patient was given 1 mg/kg of 1% methylene blue, which resolved her symptoms and normalized her methemoglobin levels to 1.1%. Ascorbic acid, in particular, has the ability to reduce the Fe3+ to the ferrous state and can be used when methylene blue cannot, such as in patients with a glucose-6-phosphate dehydrogenase (G6PDH) deficiency [3].

## Conclusions

In conclusion, methemoglobinemia is typically diagnosed through clinical findings. Partial pressure of oxygen (PO_2_) is usually normal because it measures the dissolved oxygen in the blood. When a patient is diagnosed with methemoglobinemia, treatment should be focused on providing supplemental oxygen therapy and giving the patient methylene blue as quickly as possible. Therefore, in a patient with cyanosis and without cardiac or pulmonary symptoms, methemoglobinemia should be suspected. The diagnosis is made clinically with chocolate-colored blood and an unresponsiveness to oxygen therapy. Low-dose methylene blue should be readily available, especially when topical anesthetics are used. It is also important to be aware of over-the-counter products that contain benzocaine, such as Orajel which has 20% benzocaine, as it can cause life-threatening conditions. Emergency room physicians should be aware of the clinical signs and symptoms of methemoglobinemia for rapid reversal. Improper diagnosis or treatment can result in dangerous consequences for the patient, such as seizures, coma, and eventually death.
